# Surgical removal of pulmonary flow restrictors in children with congenital heart disease: What the outcomes reveal

**DOI:** 10.1016/j.xjon.2024.11.011

**Published:** 2024-11-29

**Authors:** Raymond N. Haddad, Jamie Bentham, Carlos Pardo, Rasha Alloush, Mahmoud Al Soufi, Osama Jaber, Mohamed Kasem, Issam El Rassi

**Affiliations:** a Service de cardiologie congénitale et pédiatrique, centre de référence des malformations cardiaques congénitales complexes - M3C-Necker, Hôpital Universitaire Necker-Enfants malades, Assistance Publique-Hôpitaux de Paris, Paris, France; b Department of Congenital Cardiology, Leeds Teaching Hospitals NHS Trust, Leeds, United Kingdom; c Department of Congenital Cardiac Surgery, Gregorio Maranon University Hospital, Madrid, Spain; d Department of Pediatric Cardiology, Heart Centre of Excellence, Al Jalila Children's Speciality Hospital, Dubai, United Arab Emirates; e Department of Congenital Cardiac Surgery, Leeds Teaching Hospitals NHS Trust, Leeds, United Kingdom; f Department of Pediatric Cardiac Surgery, Heart Centre of Excellence, Al Jalila Children's Speciality Hospital, Dubai, United Arab Emirates

**Keywords:** congenital heart disease, microvascular plug, pulmonary artery band, pulmonary flow restrictor, stage-I Norwood procedure

## Abstract

**Objective:**

Pulmonary flow restrictors (PFRs) are interesting devices, but their surgical removal outcomes are poorly understood.

**Methods:**

Retrospective review of clinical data from children with bilateral PFRs who underwent device removal during follow-up surgery.

**Results:**

Thirty-four PFRs were explanted from 17 patients (41.2% boys) at a median of 2 months (interquartile range [IQR], 1.2-5.2 months) postimplantation, with a median patient age of 2.5 months (IQR, 1.6-5.8 months). One patient experienced life-threatening bilateral pulmonary artery (PA) aneurysms 2 months after PFR implantation, necessitating urgent surgery. Two PFRs were found migrated across the left PA's upper lobe branch origin. Twenty-six were removed intact, 1 in 2 fragments, and 7 piecemeal. No thrombus was noted. Neoendothelium was observed on 11 PFRs. Seven PFRs caused endothelial damage, requiring sharp and blunt dissection for removal. Six right and 4 left PA arteriotomies were patched. Hegar dilators, with median sizes of 7 mm (IQR, 6.8-8.3 mm) for right PA and 7 mm (IQR, 7-8 mm) for left PA, confirmed branch patency. At a median follow-up of 14.8 months (IQR, 10.2-18.3 months), echocardiographic maximum velocities in 13 biventricular patients and 2 awaiting future biventricular repair were 1.5 m/second (IQR, 1.4-1.7 m/second) for the left PA and 1.6 m/second (IQR, 1.4-1.7 m/second) for the right PA. One patient with deferred Norwood had normal PAs and well-positioned PFRs on prestage-II catheterization. A patient who underwent stage-II Norwood 3.2 months post-PFR implantation died from sepsis 1 month later, but post-Glenn angiogram revealed no stenosis.

**Conclusions:**

PFR removal is safe and effective. Complications are manageable, with no PA stenosis observed.

**Figure:**
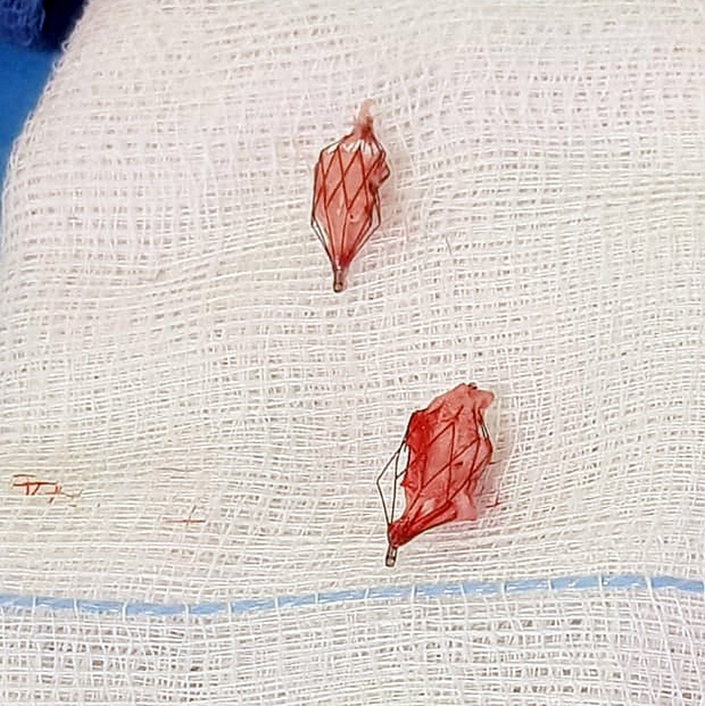
Surgical extraction of pulmonary flow restrictors.

**Central Message**

Pulmonary flow restrictors are safely removed with minimal issues, preserving pulmonary artery patency over time. Some may damage the endothelium, but outcomes remain positive with careful handling.

**Perspective**

PFRs are promising alternatives to surgical bands. This study details the removal of 34 PFRs from 17 children with CHDs, showing it is generally safe and effective, with preserved pulmonary artery patency during follow-up. Some devices cause endothelial damage, so careful handling is needed. These findings emphasize PFRs' potential to improve outcomes in complex cases.

Since its introduction in 1952, the surgical pulmonary artery band (PAB) has presented significant challenges, often necessitating reoperations due to difficulties in achieving optimal restriction.^1^, ^2^, ^3^, ^4^ Despite ongoing technical refinements, the procedure remains less than ideal. In recent years, there has been a shift toward catheter-based interventions, notably the development of pulmonary flow restrictors (PFRs).^5^, ^6^, ^7^ This innovative approach, initially demonstrated by Khan and colleagues^5^ in a growing swine model, involves repurposing the Medtronic Micro Vascular Plug (MVP) by partially removing the polytetrafluoroethylene membrane to create a partially occlusive device that allows for implantation and retrieval as needed. This method offers a less-invasive option, improving hemodynamic assessment and simplifying future interventions. A leading center in Giessen, Germany, known for its expertise in single-ventricle physiology, pioneered a purely endovascular hybrid procedure in neonates that integrated PFRs with arterial duct stenting for hypoplastic left heart syndrome (HLHS), yielding promising initial outcomes.^6^^,^^7^ Expanding beyond single-ventricle cases, we explored the application of PFRs in 2-ventricle patients with large left-to-right shunts, borderline left ventricles (LVs), and Taussig-Bing anomalies before complete repair, achieving similarly encouraging results.^8^ However, as the use of PFRs increases, concerns about potential complications, including device migration, intimal flaps, branch pulmonary artery (PA) stenosis, para-device leaks, and pulmonary overcirculation, have also risen.^9^^,^^10^ Although experience has refined strategies for managing these issues, including optimal device sizing and fenestration design, ongoing research is essential to fully understand the long-term effectiveness, potential surgical complications, and optimal use of PFRs.^11^^,^^12^ This study aims to provide new insights into the surgical outcomes of PFRs in children with congenital heart defects (CHDs), examining both the successes and challenges as we navigate this evolving field.

## Patients and Methods

### Study Design and Population

We conducted a retrospective analysis of clinical data on modified MVP implants used as PFRs in children with CHDs from September 2021 to January 2024. Our focus was on patients who underwent PFR removal during subsequent surgical palliation or repair. During this period, a total of 46 PFRs were implanted: 44 at Al Jalila Children's Specialty Hospital, 2 at Leeds Teaching Hospital, and removals included 30 PFRs at Al Jalila, 2 at Leeds, and 2 at Gregorio Marañón University Hospital. We evaluated PA patency and complications—including stenosis, distortion, neoendothelial formation, scarring, rupture, thrombosis, and aneurysm—as well as the need for subsequent PA interventions before PFR removal. Additionally, we assessed survival and PA patency following device removal. All cases were discussed and approved during multi-disciplinary team meetings before the intervention. Institutional review board approval (HCE-2024-03021000) was obtained on February 3, 2024. Informed consent for study data publication was obtained from the patient's parents.

### MVP Device and Delivery System

The MVP is a Food and Drug Administration-approved and CE-marked self-expanding mechanical occlusion device with a hexagonal nitinol wire framework. Its ovoid cylinder body tapers at both ends, with an asymmetrical polytetrafluoroethylene (PTFE) coating and radiopaque platinum markers at each end. The plug, attached to a flexible 180- or 165-cm delivery wire, detaches mechanically with anticlockwise torque. A 4-cm plastic sleeve aids in loading. Available sizes are 5.3, 6.5, 9.2, and 13 mm in diameter. The MVP-3Q and MVP-5Q have 6 and 8 covered segments, whereas the MVP-7Q and MVP-9Q have 10. The technical specifications of the MVP have been outlined previously.^8^^,^^13^

### Catheter Procedure

Catheter interventions were performed under general anesthesia, antibiotic prophylaxis, systemic heparinization, and biplane fluoroscopy. The protocols for MVP device size selection, PTFE membrane fenestration, and implantation steps have been previously outlined.^8^ We used MVP-5Q for vessels ≤4 mm, MVP-7Q for ≤6 mm, and MVP-9Q for ≤8 mm. Fenestration was created by slicing the PTFE membrane at the proximal inflow V-line with a surgical scalpel. PFRs functioned as endovascular PABs to manage pulmonary overcirculation in biventricular hearts with significant left-to-right shunts. They were implanted with or without ductal stenting during initial palliation to balance pulmonary and systemic circulation in Taussig-Bing anomalies, enhance interventricular interaction and recruit borderline LVs,^8^ and permit deferred Norwood strategy or comprehensive stage-II Norwood in HLHS variants.^7^^,^^14^ In cases of single ventricle or biventricular hearts with pulmonary overflow, we fenestrated 2 triangles in opposing diamonds on MVP-5Q, and 1 on MVP-7Q/9Q. In Taussig-Bing anomalies and borderline LVs, we fenestrated an entire diamond to address Qp/Qs mismatch while minimizing cyanosis.^8^

### Follow-up

Follow-up drug therapy included a 48-hour continuous heparin infusion (20 IU/kg/hour) and daily oral clopidogrel (0.2 mg/kg) and acetylsalicylic acid (5 mg/kg), continued until PFR removal. After hospital discharge, routine outpatient follow-ups included clinical evaluations, physical exams, saturation measurements, and transthoracic echocardiograms. Doppler velocity on implanted PFRs was assessed, and any associated general or vascular access complications were documented during these evaluations. Patients were scheduled for subsequent surgical palliation or complete repair, during which PFR removal was performed. Follow-up cardiac catheterization was conducted in patients with single ventricles before stage-II palliation or in those with borderline anatomy and physiology to plan the repair strategy. For patients with Taussig-Bing anomaly, cardiac catheterization was performed if the required information could be obtained from echocardiography and/or cardiac computed tomography scans. Patients with significant intracardiac left-to-right shunts, such as atrioventricular septal defects or ventricular septal defects, were directly referred for PFR explantation and biventricular repair.

### Surgical PFR Removal

**Figure 1:**
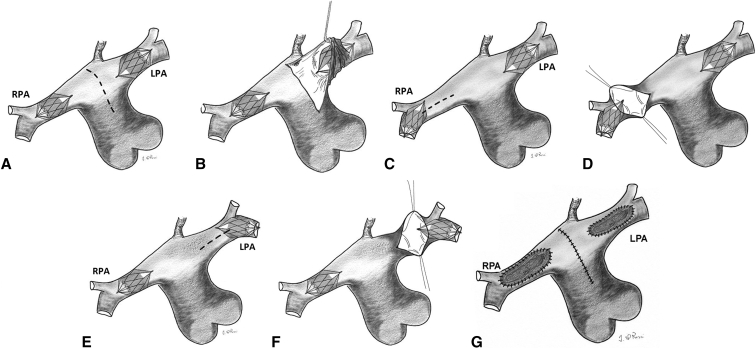
A, Extraction of pulmonary flow restrictors (*PFRs*) through main pulmonary artery (*PA*) incision (*dashed line*). B, Retraction of left and right PAs for better exposure. C, Distal migration of the device in the right PA. D, An incision on the right PA (*dashed line*) for improved PFR extraction. E, Distal device migration in the left PA. F, Anterior arteriotomy on the right PA (*dashed line*) for enhanced PFR exposure and extraction. G, Closure: main PA incision is closed directly, branch PAs are repaired with a pericardial patch. *RPA*, Right pulmonary artery; *LPA*, left pulmonary artery.

Modified MVPs were explanted during follow-up heart surgeries with cardiopulmonary bypass, such as delayed Norwood, comprehensive stage II Norwood, or biventricular repair. PFRs were carefully removed under direct vision using forceps or snare catheters, either through main PA arteriotomy or transection (Video 1), depending on the surgical plan. If needed, the longitudinal opening was extended onto the branch PA and possibly beyond the device to allow for better visualization and dissection of the PFR from the arterial wall (Figure 1, *A-D*). Special attention was given to recovering the Gore-Tex (W. L. Gore & Associates) membrane. The surgeon assessed the intima of the arterial vessels and performed repair if any vascular injury or scarring was observed. PFRs were extracted as a single piece, in 2 fragments, or piecemeal, depending on the situation, using gentle pulling and blunt dissection or a combination of blunt and sharp dissection. Thrombus formation was evaluated, and patch closure was performed when necessary (Figure 1, *E*). Branch PA patency and size were confirmed using appropriate Hegar dilators.

### Statistical Analyses

Statistical analyses were performed using SPSS version 22.0 for Macintosh (IBM-SPSS Inc). Categorical variables were reported as frequency and percentage and continuous variables were represented as median with interquartile range (IQR) as appropriate. Statistical analysis for continuous and categorical variables was conducted using the Mann-Whitney *U* test and the χ^2^ test. All reported *P* values are 2-sided.

## Results

### Patients

During the study period, a total of 46 PFRs (13 MVP-5Qs, 22 MVP-7Qs, and 11 MVP-9Qs) were implanted across 23 patients (39.1% boys), with a median age of 24 days (IQR, 10-60 days) and a median weight of 2.9 kg (IQR, 2.5-3.5 kg). The cohort included 10 patients with large intracardiac left-to-right shunts, 6 with borderline LVs, 5 with Taussig-Bing anomaly, and 2 with HLHS. Among patients with large shunts, 7 had trisomy disorders: 3 with trisomy 21, 1 with trisomy 13, and 3 with trisomy 18. For the latter 4 patients with fatal trisomies, palliation with PFRs was provided, with no further interventions planned.

### PFR Implantation

Six patients had balloon atrial septostomy: 1 with HLHS for restrictive septum and 5 with Taussig-Bing anomaly to improve mixing. No branch PA stenosis was found on the baseline angiography. The median diameter of the left PA measured 4.5 mm (IQR, 4-5.2 mm) proximally and 4.2 mm (IQR, 3.5-5.3 mm) distally. The median diameter of the right PA was 5 mm (IQR, 4.3-6 mm) at the proximal segment and 4.5 mm (IQR, 3.9-5.7 mm) at the distal segment. The intervention led to a remarkable decrease in the baseline Qp/Qs ratio, plummeting from a median of 3.1 (IQR, 2.9-3.4) to 1.3 (IQR, 1.2-1.6) (*P*  < .001). Additionally, baseline oxygen saturation saw a significant drop, falling from a median of 97% (IQR, 94%-100%) to 84% (IQR, 81%-87%) by the end of the intervention (*P* < .001). Four patients (1 with Taussig-Bing anomaly and aortic coarctation, 2 with borderline LVs, and 1 with HLHS) underwent ductal stenting, whereas 4 more recent patients (3 with borderline LVs and 1 with HLHS) were managed with prostaglandin infusion to maintain ductal patency. The median overall fluoroscopy time was 29 minutes (IQR, 20.7-38 minutes). There was no device embolization. The median noninvasive oxygen saturation was 90% (IQR, 86%-92%) at 48 hours postimplantation. No tricuspid valve injuries or vascular complications were observed.

### Short-Term Follow-up

During routine interstage catheterizations in 7 patients—6 of whom later underwent PFR explantation—we observed normal end-diastolic ventricular pressures and no signs of PFR migration, branch PA stenosis, or distortion. Control angiograms revealed no filling defects. Notably, redo catheterizations in three patients with borderline LVs confirmed their suitability for biventricular circulation. During the study period, 17 out of 23 (73.9%) patients were scheduled for subsequent surgery and PFR removal. No patients developed necrotizing enterocolitis during this period. Three patients (No. 1, No. 6, and No. 9) exhibited clinical signs of pulmonary overcirculation due to insufficient restriction and required heart failure medications until shunt closure and PFR removal. Patient No. 9 had a more complex course, which is detailed below. The last clinical follow-up was recorded at a median of 1.2 months (IQR, 0.9-5.1 months) postimplant and a median of 7 days (IQR, 4-20 days) before explantation. At this time, the median noninvasive oxygen saturation was 90% (IQR, 85%-91%), and the median maximum velocity measured by continuous-wave Doppler was 3.5 m/second (IQR, 3.2-3.9 m/second) for the left PA and 3.6 m/second (IQR, 3.1-3.8 m/second) for the right PA.

### Surgical PFR Removal

Overall, 34 PFRs (9 MVP-5Qs, 15 MVP-7Qs, and 10 MVP-9Qs) were surgically explanted at a median of 2 months (IQR, 1.2-5.2 months) postimplantation from 17 patients (41.2% boys), with a median age of 2.5 months (IQR, 1.6-5.8 months). Five patients had large intracardiac left-to-right shunts, 5 had borderline LVs, 5 had Taussig-Bing anomaly, and 3 had HLHS (see Table E1).

In 16 out of 17 patients, PFR removal was uneventfully performed as scheduled at a median of 1.8 months postimplantation (IQR, 1.2-5.6 months, range, 1-17.4 months), during biventricular repair in 12 patients, Norwood-like palliation in 2 patients with potential for future biventricular repair, stage-II Norwood in 1 patient, and deferred Norwood in 1 patient. In 2 out of 16 patients (patient No. 1 and No. 2), PFR explantation had to be delayed. For patient No. 1, the delay was 17.4 months postimplantation due to digestive issues and failure to grow despite optimal pulmonary overcirculation management. For patient No. 2, the delay was 14.7 months postimplantation due to lost follow-up and logistical overseas travel challenges. In those 2 patients, device removal was performed via PA arteriotomy without any technical difficulties.

Patient No. 9 developed febrile respiratory distress post-PFR implant, leading to intensive care unit admission. Although she initially improved, she was readmitted with a respiratory syncytial virus infection that progressed to bilateral pneumonic consolidation, requiring broad-spectrum antibiotics. After lung recovery, she underwent duct ligation via left lateral thoracotomy due to persistent signs of pulmonary overcirculation. Ten days later, bloody tracheal secretions and decreased hemoglobin levels prompted an urgent chest computed tomography scan, which revealed bilateral PFR-based PA aneurysmal dilation. Bronchoscopy showed pulsatile compression of the right intermediate bronchus and left upper lobe bronchus with a slit-like opening, leading to urgent surgery 2 months postimplant to remove the PFRs, repair PA injuries, and address the ventricular defect and mitral cleft. A 25-mm false aneurysm was found on the right PA, and the embedded PFR was removed intact. The 35-mm left PA aneurysm was opened anteriorly, and the distorted left PFR was removed piecemeal. Postsurgery, the infant required extracorporeal support for 2 weeks and targeted antibiotics until full recovery.

Two left-sided PFRs were found to have migrated across the origin of the left PA's first upper lobe branch. Overall, 24 out of 34 (70.6%) PFRs were successfully retrieved through main PA arteriotomy without complications. Branch PA arteriotomies were performed in 7 patients: 6 on the right side and 4 on the left. Bilateral PA arteriotomies were mandatory in aforementioned patient No. 9 due to complicated PA aneurysms. One of the 2 migrated left-sided PFRs was considered clinically significant, requiring a left PA arteriotomy for removal. One right PA arteriotomy was performed electively in patient No. 14 after an inadvertent laceration during PFR removal. Although the laceration could have been addressed during the Glenn anastomosis, the surgeon chose a small patch for a better aesthetic result. The remaining 6 arteriotomies involved endothelized PFRs. Thirty-two PFRs were removed using forceps under direct vision, and 2 PFRs were taken out with a snare catheter. Out of 34 PFRs removed, 26 came out whole, in 1 piece, 1 in 2 fragments, and 7 were removed piecemeal. PFRs removed in more than 1 piece occurred exclusively in the first third of our patient cohort. No signs of thrombus formation were observed on any of the retrieved devices.

Neoendothelium formation was observed in 6 patients, on 6 of 17 PFRs explanted from the right PA and 5 of 17 from the left PA. These 11 PFRs (2 MVP-5Q, 3 MVP-7Q, and 6 MVP-9Q) were removed at a median of 3.7 months (IQR, 1.4-6.4 months) postimplantation. In comparison, 23 PFRs (7 MVP-5Qs, 12 MVP-7Qs, and 4 MVP-9Qs; *P* = .99) removed from 11 patients without neoendothelium were extracted at a median of 1.6 months (IQR, 1.3-3.4 months; *P* = .88). In 4 of these 6 patients with neoendothelialized PFRs, 4 right-sided and 3 left-sided PFRs caused damage to the intimal endothelial layer, necessitating meticulous sharp and blunt dissection with scissors and spatula for safe removal, performed at a median of 3.7 months (IQR, 2-6.3 months) postimplantation. Autologous pericardium was used to patch repair the 6 right PA (RPA) and 4 left PA (LPA) arteriotomies. Hegar dilators were smoothly introduced into the RPA and LPA, with median sizes of 7 mm (IQR, 6-8 mm) and 7 mm (IQR, 6-8 mm), respectively, demonstrating branch patency and size.

### Postsurgery Follow-up

**Figure 2:**
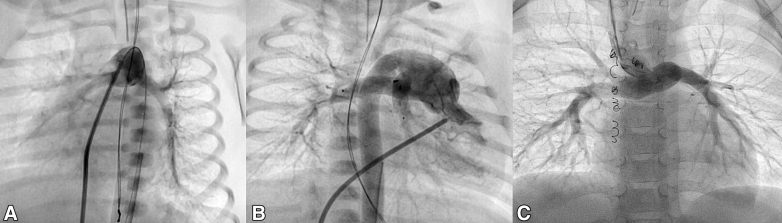
Pulmonary angiograms. A, Before pulmonary flow restrictors (*PFRs*) implantation. B, After PFR implantation. C, Post-Glenn anastomosis following PFR removal.

Following surgery, the median intensive care unit stay was 6 days (IQR 3-13 days), with median ventilation support lasting 3 days (IQR 1-8 days) and inotropic support lasting a median of 4 days (IQR 3-10 days). At hospital discharge, the median maximum velocity measured by continuous-wave Doppler was 1.5 m/second (IQR 1.4-1.7 m/second) for the LPA and 1.6 m/second (IQR 1.4-1.7 m/second) for the RPA. For the 13 patients with biventricular hearts and 2 aiming for future biventricular repair, the follow-up was uneventful, with no need for exit angiography or redo catheterizations. Following a median outpatient follow-up of 14.8 months (IQR, 10.2-18.3 months), the PAs in these 15 patients demonstrated patency with no increase in the recorded ultrasound velocities. Patient No. 15 with deferred Norwood underwent a prestage-II cardiac catheterization 6 months after PFR implantation, which showed well-positioned PFRs without PA distortion or stenosis. Patient No.14 had stage-II Norwood 3.2 months after PFR implantation. He died 1 month after surgery from severe necrotizing enterocolitis-associated sepsis. He had a post-Glenn angiogram which showed no branch PA stenosis (Figure 2).

### Nonexplanted PFR Follow-up

One patient is alive 1 month post-PFR implantation and is scheduled for surgical PFR removal and biventricular repair. One patient with trisomy 13 and another with trisomy 18 are alive at 14 and 1.5 months postprocedure, with no further interventions planned. There were 3 late deaths before the PFR explant. One patient with Shone's complex variant and a borderline LV died 14.8 months after PFR implantation and ductal stenting due to severe Gram-negative sepsis while he was in preparation for future biventricular repair. This patient underwent redo catheterization 7.4 months post-PFR implantation for redo ductal stenting, during which the PA angiogram showed well-implanted PFRs without distortion or stenosis. Two patients with trisomy 18 died at 1 and 6.8 months after the procedure from non-cardiac causes (see Table E2).

## Discussion

This study explores the understudied area of PFR removal in children with CHD, focusing on its safety, effectiveness, and outcomes. Our retrospective analysis of 17 children provides key insights into the challenges and successes of the procedure.

According to the Boston Children's Hospital team, PFRs are transforming the management of CHDs requiring temporary PA blood flow modulation,^15^ and reviving endovascular PAB, which had been overshadowed by early surgical repairs.^6^, ^7^, ^8^ Complex neonatal surgeries, despite their risks, have often been prioritized for addressing multiple anomalies in small newborn infants.^16^, ^17^, ^18^ Inspired by Schranz and colleagues,^6^, ^7^ Wernovsky and colleagues^19^ recently proposed rapid bilateral PAB for neonates with HLHS as a developmentally focused alternative to deferring the Norwood procedure. They highlighted the potential benefits of catheter-based PFRs while acknowledging uncertainties regarding surgical removal.

Innovative MVP-based PFR devices promise to delay complex surgeries until infants reach a higher weight, reducing early morbidity and shortening intensive care unit and hospital stays.^8^^,^^12^^,^^15^ However, concerns persist about their use. Nageotte and colleagues^9^ reported distal migration in 5 of 6 patients with HLHS variants, often into the right PA, despite device oversizing. One patient developed left PA stenosis after 96 days, requiring stenting. Pulmonary overcirculation was noted in 4 of 6 patients, necessitating further interventions. Sandoval and colleagues^10^ also questioned PFR efficacy in restrictive single-ventricle physiology, despite Warren and colleagues'^14^ positive outcomes.

The customization of the MVP is promising but still in its initial stages, with limited reports urging caution. These experiences provide valuable complementary insights. We previously shared views on device sizing and fenestration methods.^8^ Ballooning the thin PTFE membrane, as noted by Nageotte and colleagues,^9^ and crossing PFRs for pressure readings are debatable practices. Proper oversizing is crucial for stable implantation, as excessive oversizing can cause paradevice leaks and reduce PFR effectiveness.^13^ Intraprocedural systolic-diastolic Doppler patterns help prevent pulmonary overcirculation and confirm adequate flow restriction before exiting the catheterization lab.^12^ Hemodynamics in sedated patients can alter PFR effects, and cardiovascular comedications may influence overflow conditions. As Schraz^12^ highlights, interventional measures, including anesthesia and cardiac support, should be considered during and after the procedure.

Most technical and short-term catheter-related issues have been well-debated in the literature. The focus now shifts to surgical outcomes of PFRs after removal and whether or not these devices may cause new lesions requiring future intervention.

From a technical standpoint, our surgeons found PFR removal to be straightforward. After sternotomy, PFRs were not used to limit pulmonary blood flow during cardiopulmonary bypass; instead, additional clamps or snares were applied proximally on the branch PAs to prevent distortion or fracture of the devices, which could complicate removal. Typically, the main PA is opened to assess the devices' position and adhesion to the arterial wall. The proximal end of PFRs is usually visible in the PA lumen, facilitating removal. PFRs migrating distally beyond the first branch are harder to locate but can often be accessed through the main PA opening with proper suction and exposure. Two left-sided PFRs migrated across the origin of the left PA's first upper lobe branch. One was removed easily using a snare catheter, whereas the other was deemed clinically significant, requiring a longitudinal arteriotomy for its removal. Although North American groups report migration rates above 30%,^9^^,^^15^ we believe our device size selection protocol^8^ and the use of a steerable microcatheter^20^ have been instrumental in reducing migration rates in our cohort.

For long-standing PFRs, longitudinal branch openings and patching were often necessary. Although neoendothelium formation was not statistically linked to implantation duration or device size, neoendothelialized PFRs were removed, on average, 2 months later than those without neoendothelium, which were removed within 8 weeks. PFRs in place for <8 weeks can typically be removed intact through the main PA opening with gentle pulling and blunt dissection, although the distal segment may adhere more firmly due to the absence of the Gore-Tex membrane. For PFRs in situ for more than 2 months, sharper dissection is required, which can remove the intima, as seen in 4 of 6 patients with neoendothelialized PFRs. Long-standing PFRs were often extracted in pieces due to the fragile nitinol wire framework, leaving small fragments (2-3 mm) that are unlikely to cause harm. Although we did not experience any instances of Gore-Tex membrane being left behind or related adverse events, recovering the membrane is essential in case of fracture. We acknowledge that a learning curve was necessary to prevent branch arteriotomies and device fractures in endothelized devices. Initially, our limited experience with MVP-based PFR fragility led us to open PA branches for safe removal. However, as our technique improved, we successfully extracted most devices intact from the trunk. Surgeons mastered this process through blunt and sharp dissection, learning to handle the device by its delivery tip and gently push the arterial wall away to avoid contact with the delicate PFR.

Postremoval, the area was inspected for intimal flaps or debris; the arterial wall may show a honeycomb imprint of the mesh without any observed consequences. We observed no thrombus formation on retrieved devices, indicating that dual antiplatelet therapy effectively prevented this complication.

We previously reported a serious PFR complication in a small infant.^21^ A viral respiratory infection, complicated by bacterial pneumonia, likely caused inflammation, lung damage, and weakened PA walls, resulting in bilateral aneurysms.^22^ Bacteremia likely worsened PA wall weakness, with foreign material, bacteremia, and flow turbulence contributing to endothelial injury. Although not specific to MVPs in branch PAs, this complication emphasizes the need for stricter safety standards for custom devices in neonates with single ventricles. The risk of PA tract infections, especially during interventions, may be higher in this population.

In conclusion, PFRs were removed safely with minimal complications. Most were extracted intact, with a few requiring piecemeal removal due to endothelial damage and the learning curve. Neoendothelium formation was observed without thrombus. Postremoval assessments showed good PA branch patency and favorable hemodynamics at discharge for most patients, without the need for further interventions. Although 1 patient experienced severe complications unrelated to PA stenosis, the clinical course was largely uneventful. These results support the efficacy of PFRs for CHDs and highlight the importance of precise surgical technique and follow-up.

### Limitations

Study limitations include small sample size, patient heterogeneity, retrospective design, and relatively short follow-up. The initial learning curve associated with any novel procedure may influence outcomes. However, the study offers valuable insights into the potential of this innovative approach.

## Conclusions

MVP-based PFRs can be effectively removed with minimal complications. Although long-standing PFRs may cause some endothelial damage and require varied removal techniques, outcomes show favorable PA patency. Follow-up indicates positive results in explanted patients, with no significant issues reported in those awaiting future repairs.

## Conflict of Interest Statement

The authors reported no conflicts of interest.

The *Journal* policy requires editors and reviewers to disclose conflicts of interest and to decline handling or reviewing manuscripts for which they may have a conflict of interest. The editors and reviewers of this article have no conflicts of interest.

## Figures and Tables

**Table E1:** Clinical and procedural characteristics of patients with explanted pulmonary flow restrictor (PFR)

PN	G	W∗	A∗	Diagnosis	Left PFR	Right PFR	DTE	LPA LA	Left PFR NF	LPA HDS	RPA LA	Right PFR NF	RPA HDS	FU	Final status	Vmax LPA	Vmax RPA
1	F	3.6	73	Borderline LV: Unbalanced AVSD, HAA, Ao CoA, Apical VSD, s/p surgical PAB, and Ao CoA repair	9Q	9Q	17.4	No	No	9	No	No	9	19.9	BiV	1.4	1.5
2	F	4.0	29	Taussig-Bing anomaly, Ao CoA	7Q	7Q	14.7	No	No	8	No	No	8	18.3	BiV	1.7	2.1
3	F	2.8	13	Borderline LV: Subaortic stenosis, Ao CoA, sub-pulmonary VSD	5Q	7Q	5.2	Yes	Yes +Damaged Endothelium	9	Yes	Yes + Damaged Endothelium	9	25.7	BiV	1.7	1.8
6	M	5.0	87	Large VSD	9Q	9Q	1.0	No	Yes	9	No	Yes	9	25.0	BiV	1.7	1.6
7	M	3.6	72	Complete AVSD	9Q	9Q	6.8	No	Yes	7	Yes	Yes	7	17.4	BiV	1.4	1.2
8	F	2.9	13	Taussig-Bing anomaly	5Q	7Q	2.0	No	No	7	No	No	7	18.4	BiV	1.9	1.6
9	F	3.4	63	Large VSD, PDA, ASD, mitral cleft, T21	9Q	9Q	2.2	Yes – aneurysm	Yes + damaged endothelium	6	Yes – false aneurysm	Yes + damaged endothelium	7	17.9	BiV	1.2	1.1
12	F	2.4	33	Complete AVSD, T21	5Q	5Q	4.7	No	No	7	No	No	7	14.6	BiV	1.5	1.4
13	F	3.9	28	Complete AVSD, PDA, T21 s/p PDA ligation	7Q	5Q	9.6	Yes	Yes + damaged endothelium	8	Yes	Yes + damaged endothelium	9	9.7	BiV	1.3	1.3
14	F	2.8	5	HLHS	5Q	7Q	2.2	No	No	6	Yes	No	6	1	Dead	1.7	1.9
15	M	4.6	5	HLHS	5Q	7Q	1.6	Yes	No	6	No	No	8	15.1	UniV	1.6	1.7
16	F	2.6	16	Borderline LV: Unbalanced AVSD, HAA, Ao CoA	7Q	7Q	1.4	No	No	7	No	No	8	11.7	BiV	1.5	1.0
17	M	3.4	14	Taussig-Bing anomaly	7Q	9Q	1.1	No	No	7	No	No	7	11.9	BiV	1.7	1.4
18	M	3.3	4	Taussig-Bing anomaly	7Q	7Q	1.1	No	No	6	Yes	Yes + damaged endothelium	6	10.4	Aiming for BiV	1.4	1.7
19	M	2.5	13	Taussig-Bing anomaly	7Q	7Q	1.2	No	No	6	No	No	6	8.8	BiV	1.5	1.7
20	M	2.7	3	Borderline LV: Type B IAA, aortic valve and outflow hypoplasia, large muscular VSD	7Q	9Q	1.5	No	No	6	No	No	6	5.9	Aiming for BiV	1.5	1.7
21	F	3.2	7	Borderline LV: Type A IAA, aortic valve hypoplasia, membranous VSD	5Q	5Q	1.2	No	No	6	No	No	6	2.4	BiV	1.5	1.7

*PN*, Patient number; *G*, gender; *W*, weight (in kilograms); *A*, age (in days); *DTE*, delay to explantation (in months); *LPA*, left pulmonary artery; *LA*, longitudinal arteriotomy; *NF*, neoendothelium formation; *HDS*, Hegar dilator size; *RPA*, right pulmonary artery; *FU*, follow-up (in months) from PFR removal date; *Vmax*, maximum velocity measured by continuous-wave Doppler (m/second) at last recorded follow-up; *F*, female; *LV*, left ventricle; *AVSD*, atrioventricular septal defect; *HAA*, hypoplastic aortic arch; *Ao CoA*, aortic coarctation; *VSD*, ventricular septal defect; *s/p*, status post; *PAB*, pulmonary artery band; *BiV*, biventricular; *M*, male; *PDA*, patent ductus arteriosus; *ASD*, atrial septal defect; *T21*, trisomy 21; *HLHS*, hypoplastic left heart syndrome; *UniV*, univentricular; *IAA*, interrupted aortic arch.

∗ At the time of PFR implantation.

**Table E2:** Clinical and procedural characteristics of patients with nonexplanted pulmonary flow restrictors (PFRs)

PN	G	W∗	A∗	Diagnosis	Left PFR	Right PFR	FU	Final status	Vmax LPA	Vmax RPA
4	F	2.7	59	Large VSD, PDA, ASD, T18	7Q	7Q	6.8	Dead: Noncardiac causes	3.5	3.6
5	M	3.2	36	Borderline LV: aortic stenosis, aortic coarctation, no VSD, s/p aortic valve ballooning, and atrial septostomy	7Q	7Q	14.8	Dead: Gram-negative sepsis	4.1	4
10	F	2.2	71	Multiple VSD, PDA, T18	7Q	9Q	1	Dead: Noncardiac causes	2.9	2.8
11	F	1.9	62	Large VSD, PDA, T13	5Q	5Q	14	Alive: No further intervention planned	3.3	3.2
22	F	2.1	0	Large VSD, T18	7Q	5Q	1.5	Alive: No further intervention planned	4.1	2.9
23	M	2.4	24	Large VSD, PDA	5Q	7Q	1	Alive: Aiming for BiV repair	3.8	3.7

*PN*, Patient number; *G*, gender; *W*, weight (in kilograms); *A*, age (in days); *FU*, follow-up (in months) from PFR implantation date; *Vmax*, maximum velocity measured by continuous-wave Doppler (in m/second) at last recorded follow-up; *LPA*, left pulmonary artery; *RPA*, right pulmonary artery; *F*, female; *VSD*, ventricular septal defect; *PDA*, patent ductus arteriosus; *ASD*, atrial septal defect; *T18*, trisomy 18; *M*, male; *LV*, left ventricle; *s/p*, status post; *T13*, trisomy 13; *BiV*, biventricular.

∗ At the time of PFR implantation.

## Supplementary Data

Video 1 Surgical pulmonary flow restrictors extraction 1.5 months postimplantation, through transected main pulmonary artery. Video available at: https://www.jtcvs.org/article/S2666-2736(24)00411-X/fulltext.
