# Supplementary figures and images for: Surgical removal of pulmonary flow restrictors in children with congenital heart disease: What the outcomes reveal
Source: JTCVS Open. 2024 Nov 29;23:235–44. doi: 10.1016/j.xjon.2024.11.011 (PMC11883757; doi:10.1016/j.xjon.2024.11.011)

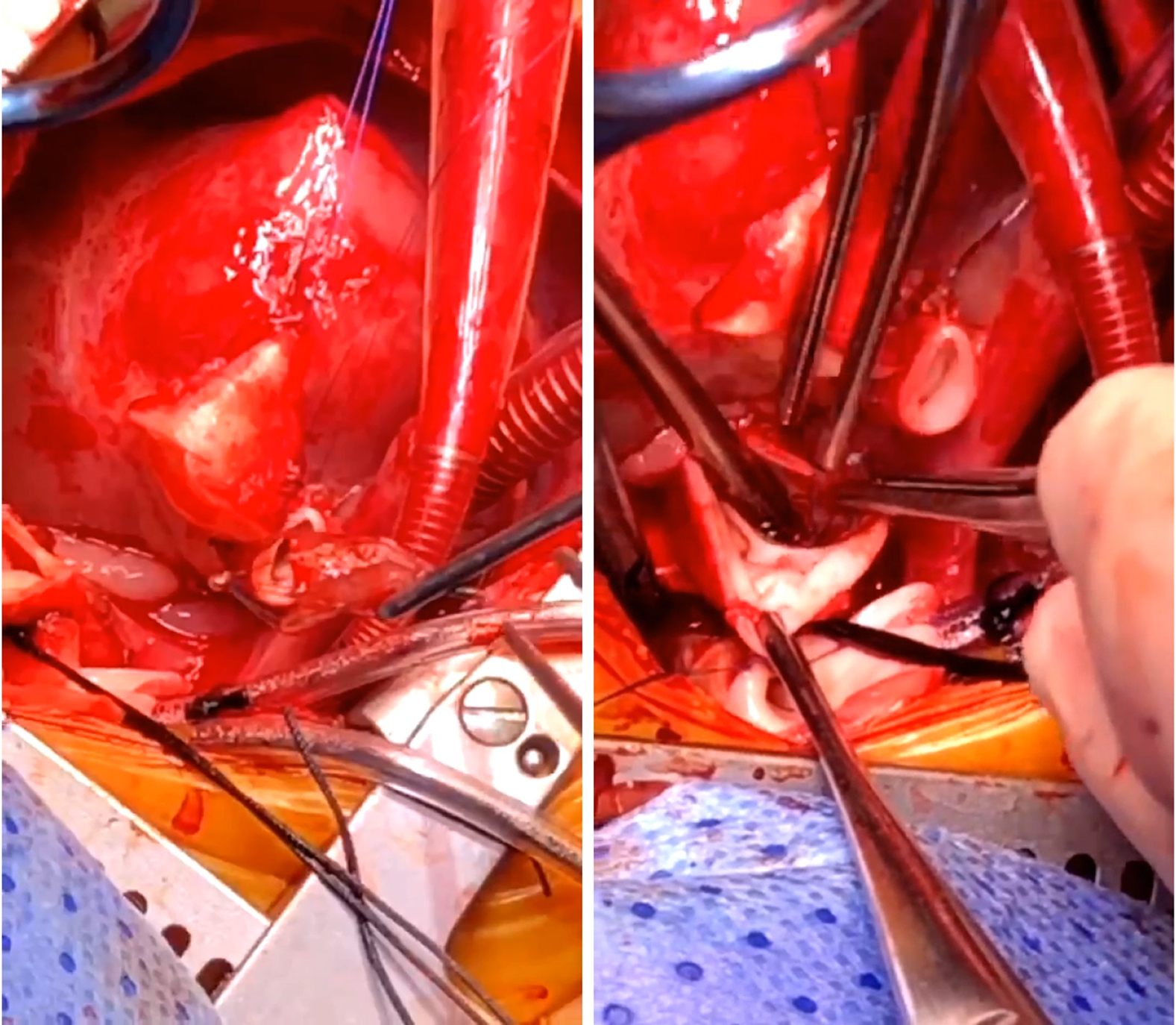

Supplement: Video 1 — Surgical pulmonary flow restrictors extraction 1.5 months postimplantation, through transected main pulmonary artery. Video available at: https://www.jtcvs.org/article/S2666-2736(24)00411-X/fulltext. [file fx2.jpg]
